# Treatment of scoliosis in patients affected with Prader-Willi syndrome using various techniques

**DOI:** 10.1186/1748-7161-5-11

**Published:** 2010-06-15

**Authors:** Tiziana Greggi, Konstantinos Martikos, Francesco Lolli, Georgios Bakaloudis, Mario Di Silvestre, Alfredo Cioni, Giovanni Barbanti Bròdano, Stefano Giacomini

**Affiliations:** 1 Spinal Deformity Surgery Department, Rizzoli Orthopaedic Institute, Via Pupilli, 1, 40126 Bologna, Italy

## Abstract

**Background:**

The incidence of spinal deformity in children with Prader-Willi syndrome (PWS) is high, with 86% of these patients found to have a significant structural scoliosis; however, there are very few case reports describing surgical treatment for this deformity.

**Methods:**

The authors reviewed a case series consisting of 6 patients who underwent spine surgery for scoliosis. Children's mean age at index surgery was 12 years and 10 months (range, 10 to 15 yrs). Clinical evaluation revealed the typical phenotypic features of the PWS in all of the patients; 4 subjects had a karyotype confirmation of PWS. Major structural curves showed preoperative mean Cobb angles of 80.8° (range, 65° to 96°). Hybrid instrumentation with sublaminar wires, hooks and screws was used in the first 2 patients, while the remaining 4 were treated with titanium pedicle screw constructs.

**Results:**

The mean clinical and radiological follow-up was 3 years and 10 months (range, 2 years to 9 years). Major complication rate was 50%. One patient who developed a major intraoperative complication (paraparesis) prevented spinal fusion to be obtained: the neurologic deficit resolved completely after instrumentation removal. Solid arthrodesis and deformity correction in both coronal and sagittal plane was, however, achieved in the other 5 cases and no significant curve progression was observed at follow-up. Another major short-term complication was encountered 3 months after surgery in a patient who experienced the detachment of a distally located rod and required correction through revision surgery and caudal extension by one level. Cervico-thoracic kyphosis was seen in 1 patient who did not require revision surgery.

**Conclusions:**

Spine reconstructive surgery in patients with PWS is rare and highly demanding.

The best method of reconstruction is posterior multilevel pedicle screw fixation. Moreover, even with modern techniques, the risk of complications is still high. These new techniques, however, have shown to improve the postoperative course by allowing for immediate mobilization without any brace or cast. The use of the growing rod techniques, requiring repeated surgeries, should be carefully evaluated in each single case.

## Background

The Prader-Willi syndrome (PWS) is a rare and complex genetic disorder, first described by Prader et al back in 1956 [1], related to deletion (70% of the cases), duplication (25% of the cases) or other types of alteration of the paternal copy of chromosome 15, q 11.2 - 13 [2,3]. The syndrome affects 1 in 15000 newborns, with a 3 to 1 male-female ratio. The various concomitant pathological features of this syndrome that define its consensus diagnostic criteria as established by Holm et al in 1993 [4] include muscular hypotonia, which tends to improve during the first year of life, low stature, narrow forehead, childhood obesity, ligamentous hyperlaxity, fragile hair, light complexion, prominent lower lip, hypogonadism, diabetes mellitus, delay in psychomotor development, intellectual deficit, squint, myopia, sleeping disorders with sleep apnea, clinodactyly, valgus knee with instability and osteoporosis. Severe deformities of the spine in both frontal and sagittal planes are also frequently observed.

The reported prevalence of scoliosis varies from 45% to 86%, whereas the hyperkyphotic deformity can be observed in nearly 40% of the patients [5,7,8]. Spinal diseases are considered supportive criteria with no direct impact on the diagnostic score established by Holm et al. The syndrome is associated with growth hormone deficiency (GHD). Although the pharmacological therapy with growth hormone (GH) provides satisfactory results, its impact on the progression of spinal deformities still remains controversial [5,6]. Scoliosis is reported to require active treatment in 15% to 20% of the cases and to behave as an idiopathic scoliosis, with a high risk of progression during adolescence [1,7].

The various clinical and pathological conditions with onset during the paediatric and early adolescent age, and affecting the general aspect and health status, can change or delay treatment of spinal deformities and subsequently entail negative consequences in terms of life quality and expectation. Inadequate or delayed treatment of spinal deformities can jeopardize the patient's respiratory and cardiocirculatory "compliance", which is already severely compromised by the other notable phenotypic aspects of the syndrome.

## Methods

The current series consists of 6 patients (5 males and 1 female) affected with Prader-Willi syndrome, who underwent spine surgery for scoliosis between 1998 and 2009 at the authors' Spine Surgery Division (Table 1). All of the patients presented with the typical phenotype of the Prader-Willi syndrome: 4 of them had karyotype confirmation of Prader-Willi syndrome, 1 presented with a negative genetic test for the Prader-Willi syndrome, whereas the remaining case never underwent DNA laboratory tests. All children had been diagnosed with scoliosis in pre-adolescent age.

**Table 1:** Surgical procedures and postoperative data

	First surgery year	Age at first surgery	Surgery description	Surgery duration	Blood losses during surgery	Hospital stay after surgery	Preop Cobb angle of primary scoliosis	Postop Cobb angle of primary scoliosis	Correction rate
**B.S.F**.	1998	14 yrs	T4-L4 posterior arthrodesis using **hybrid instrumentation **with sublaminar wires, hooks and a couple of distally-inserted pedicle screws	3 hrs	1000 ml	10 days	79°	15°	81%
**M.S**.	1998	10 yrs	T2-L2 posterior arthrodesis using **hybrid instrumentation**: proximally-inserted hooks and a couple of distally-inserted screws in L1 and L2, as well as 2 DTTs	4 hrs	3000 ml	17 days	65°	Instrumentation removal	-
**C.F**.	2005	12 yrs	T4-L4 posterior arthrodesis using only **titanium pedicle screw instrumentation**	6 hrs	1500 ml	9 days	95°	33°	65%
**P.L**.	2006	11 yrs	T3-L4 growing rod instrumentation using only **titanium pedicle screws; **final fusion after 18 months	5 hrs	2000 ml	10 days	96°	32°	63%
**E.H.J**.	2007	15 yrs	T3-L2 posterior arthrodesis using only **titanium pedicle screw instrumentation**	6 hrs	1000 ml	10 days	75°	35°	53%
**O.M**.	2007	15 yrs	T5-L3 posterior arthrodesis using only **titanium pedicle screw instrumentation**	6 hrs	2000 ml	10 days	80°	50°	38%

Mean age at index surgery was 12 years and 10 months (range, 10 to 15 yrs); the primary scoliotic curve ranged from 65° to 96°, and the mean thoracic kyphosis was 62.3°. Thoracic kyphosis was within normal values only in 1 patient (31°), while it ranged from 61° to 82°in the remaining 5. At the time of surgery, mean skeletal maturity according to Risser sign was 2.3 (range, 0 to 4): this finding, associated with the above mentioned radiographic data, confirms the rapid progression of the deformity right from early age. Indication for surgery was, therefore, severe progressive scoliosis during the adolescent growth spurt and was associated with hyperkyphosis in 5 cases. Hybrid instrumentation with sublaminar wires, hooks and screws was used in the first 2 patients while the remaining 4 were treated with titanium pedicle screw instrumentation. In all of the case, bone graft used for arthrodesis was homologous fresh-frozen banked bone, to avoid painful grafting from the patients' iliac crest. The mean clinical and radiological follow-up was 3 years and 10 months (range, 2 years to 9 years).

Some difficulty in vascular access was encountered in two patients by anesthesia, and bladder catheterization was also different in another two patients due to hypospadias. The American Society of Anesthesiologists (ASA) mean risk index was 2 (range, 1 to 3); the spirometry test revealed severe restrictive pulmonary deficit in 4 patients, and reduced values in another 2. None of the patients presented with any neurological deficits. One child had past history of fever convulsions until the age of 6. No severe cardiologic diseases were seen, apart from a mild mitral valve prolapse in 1 subject. Mean Body Mass Index (BMI) was 29.5 (range, 21 to 42). The mean weight and height were 60.8 kg (range, 35 to 99) and 1.4 m (range, 1.05 to 1.73), respectively. Four children had received GH treatment in the past; none of them presented with pathological hyperglycemia.

All patients underwent total MRI spinal examination before surgery that excluded intraspinal anomalies or dysraphysm.

Full-time brace was applied for at least 2 years before surgery in 3 cases, though with little impact on curve progression.

## Case Reports

(in chronological order of surgical procedure)

### Case 1

B.S.F. (male, born in 1984) The first patient treated at the authors' Spine Surgery Division had the typical P.W. phenotype, though not confirmed by the genetic laboratory tests. By the age of 14, skeletal maturity was 3 according to the Risser test and the patient presented with a single wide left convex thoracolumbar curve of 79°, with curve apex in L1, and no congenital vertebral anomalies. Thoracic kyphosis was 82° and the sagittal cervico-sacral plumb line was + 10.6 cm. The boy had not followed any proper conservative treatment before, as he could not tolerate any kind of brace. The other concomitant pathologic conditions observed, were renal hypotrophy, gastroesophageal reflux and urinary incontinence. Spirometry revealed severe pulmonary deficit: Forced Vital Capacity (FVC) 37% and Forced Expiratory Volume (FEV) -1 44%. Spinal arthrodesis from T4 to L4 was performed in 1998 at the age of 14 using hybrid instrumentation with sublaminar wires, hooks and distally inserted pedicle screws. Postoperatively, the Cobb angle was 15°, the thoracic kyphosis 62° and the sagittal plumb line decreased to + 2.4 cm. There were no complications during and after the 3-hour surgery; intraoperative blood loss was 1000 ml. The pharmaceutical painkiller administered was only paracetamol for the first 3 postoperative days. No brace was applied after surgery and at a 9-year follow-up, correction was stable. Three years after surgery, a mild lateral deviation of the cervical spine was seen on the AP plane: radiographic measurements showed an anteroposterior cervico-sacral plumb line deviation by -4.1 cm, still unchanged at last follow-up.

### Case 2

M.S. (male, born in 1988) The patient had the positive phenotypic and karyotypic findings consistent with P.W. syndrome. He had remarkable obesity with a BMI of 41.5, and a past history of frequent bronchopulmonary infections. Spirometry revealed a severe pulmonary deficit: FVC 43% and FEV-1 47%. The patient had received no brace treatment before 1998: his deformity consisted of a structural right convex thoracic scoliosis of 65° with another structural left convex lumbar curve of 55° and thoracic kyphosis of 60°. Skeletal maturity according to Risser sign was still 0. At the age of 10, the patient underwent spine surgery: spinal arthrodesis was performed from T2 to L2 using hybrid instrumentation with hooks at proximal levels and pedicle screws at two lumbar levels. During surgery a major complication occurred: at the end of the instrumentation procedure, the intraoperative wake-up procedure revealed transient paraparesis (grade B, Asia Impairment Scale), requiring total instrumentation removal. At final wake-up, symptoms had partially resolved and improvement was observed during the following 76 hours (grade D, Asia Impairment Scale). Surgery lasted 4 hours and blood loss was 3000 ml. Four days after the surgical procedure, severe dyspnoea occurred with oxygen saturation deficit (80%), successfully treated with prompt medical measures. Despite full-time brace treatment for 5 months after surgery, a severe progression of scoliosis and thoracic kyphosis was seen at a 3-year follow-up. From the neurologic point of view, there was a near complete recovery of motor and sensory function and with ambulatory capacity. However, there remains sphincter disturbance.

### Case 3

**Figure 1:**
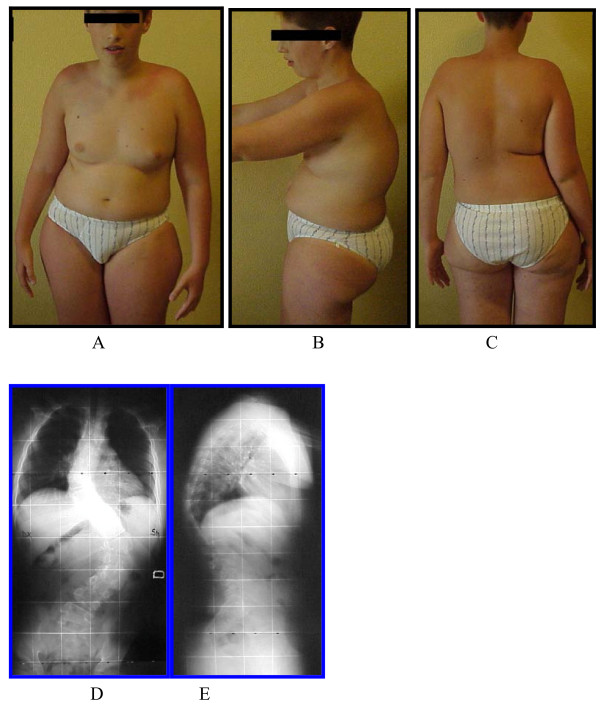
**Preoperative clinical and radiographic picture**. C.M., male, born in 1993, operated on in 2005; severe kyphoscoliosis in PWS.

**Figure 2:**
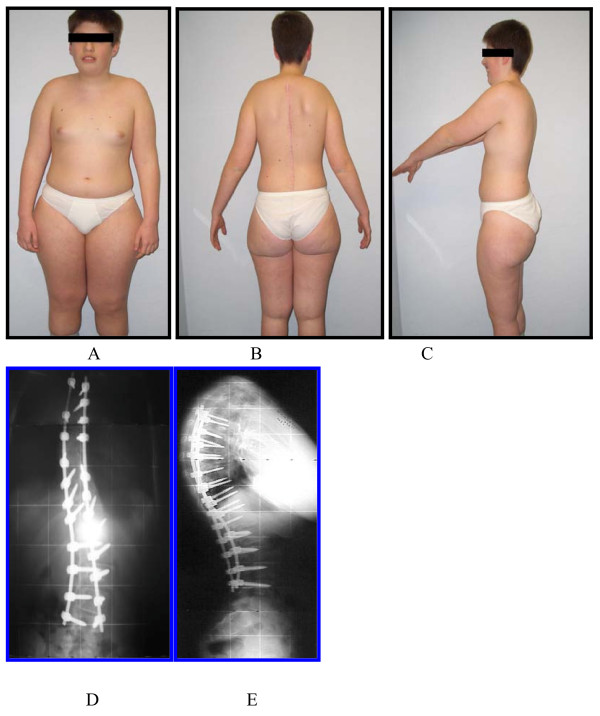
**Clinical and radiographic picture at a 2-year follow-up**. C.M., clinical and radiographic follow-up in 2007: T4-L4 arthrodesis using only pedicle screws. A notable deformity correction was achieved and is now visible.

C.M. (male, born in 1993) The patient presented the typical clinical aspects of the P.W. syndrome, although he had never undergone genetic testing for P.W. He had a BMI of 29 and had received GH during paediatric age. By the age of 2, he had already undergone surgery for cryptorchidism. At the time of the spine surgery, despite the full-time brace worn for 3 consecutive years, scoliosis had evolved into a left convex lumbar curve of 95°, with a compensating right thoracic curve of 55° and thoracic kyphosis of 65° [Figures 1a, 1b, 1c, 1d and 1e]. In 2005, at the age of 12 and with a Risser sign of 2, posterior T4 - L4 spinal arthrodesis was performed using pedicle screws exclusively: the procedure lasted 6 hours; blood loss was 1500 ml. Postoperative radiographic values showed the primary curve had decreased to 33°, the secondary curve to 30° and thoracic kyphosis to 51°. No short-term complications were observed; postoperative pain was successfully controlled with a relatively small amount of minor opioids and Non-Steroidal Anti-Inflammatory Drugs (NSAIDs) administered for 4 days only. No brace was applied after hospital discharge and curve correction was maintained at 4 years postop. No long-term complications were reported, although the patient had pain with ambulation due to degenerative valgus knee deformity [Figures 2a, 2b, 2c, 2d and 2e].

### Case 4

P.L. (female, born in 1995) The patient underwent surgery for scoliosis at the authors' Spine Surgery Division in 2006, at the age of 11, while the Risser sign was 2. Both clinical and genetic evaluation confirmed the P.W. syndrome. Muscular hypotonia, hypogonadism, strabismus, myopia and glucose intolerance were registered in the authors' medical archive. Preoperative radiographic assessment of the spine revealed a primary right thoracic curve of 96°, another structured left lumbar curve of 90° and a thoracic kyphosis of 61°. A growing rod instrumentation was applied from T3 to L3 using pedicle screws at L3, L2, T9, T8, T6 and T3, and laminar hooks at T4 and T3. The thoracic curve decreased to 78°, the lumbar curve to 72° and the thoracic kyphosis to 55°. The child wore a Lyonnaise orthosis 16 hours a day for 2 months and tolerated it well. One year after surgery, the curves returned to the preoperative values and an increase in the cervico-thoracic kyphosis was observed without proximal instrumentation mobilization. No lengthening was performed, as the growing rod technique would require, since it was not possible to reach an agreement with the patient's parents.In December 2007, 18 months after the first surgery, a second surgical procedure was performed to achieve final arthrodesis.

The upper part of the instrumentation was left intact while screws were applied at all levels from T9 to L4. Surgery lasted 5 hours and blood loss was 2000 ml. No short-term complications were observed. A 46° correction at thoracic level and a 36° correction at lumbar level were achieved, while thoracic kyphosis decreased to 48°. Postoperatively, a cast orthosis was applied for 2 months. No complications were seen in the immediate postoperative period and at a clinical and radiographic follow-up of 3 years, when the correction was still stable. The child, still under GH treatment, continues to wear a Lyonnaise orthosis 14 hours a day, is able to ambulate autonomously, while the cervico-thoracic kyphosis remains stable.

### Case 5

E.H.J. (male, born in 1992) He was affected with P.W. syndrome as confirmed by clinical examination and karyotype test. Before surgery he had a BMI of 29,3 and had been previously treated with GH. He had been born prematurely and had received surgical treatment for bilateral orchiopexy in his second year of life. In addition, he had a clinical history of allergy towards many NSAIDs and digestive intolerance to milk. Spirometry revealed a severe pulmonary deficit: FVC 57% and FEV-1 62%. Full-time Milwaukee brace treatment since the age of 8 had failed to control the progression of the deformity and by the age of 15, while the Risser sign was 3, scoliosis had evolved into a structured right thoracic curve of 75° and a structured left lumbar curve of 56°, with thoracic kyphosis of 71°. In March 2007, instrumented posterior arthrodesis was performed from T3 to L2 using pedicle screws exclusively; the 6-hour surgery enabled satisfactory corrections of curves to be achieved: the thoracic curve decreased to 35°, the lumbar curve to 30° and thoracic kyphosis to 53°. Intraoperative blood loss was 1000 ml. After a 4-month brace-free period, correction was stable and no complications were registered, as further confirmed at last follow-up in March 2009. Before and after surgery, the patient's ambulation was unstable with balance difficulty due to a severe and inveterate bilateral valgus knee deformity.

### Case 6

**Figure 3:**
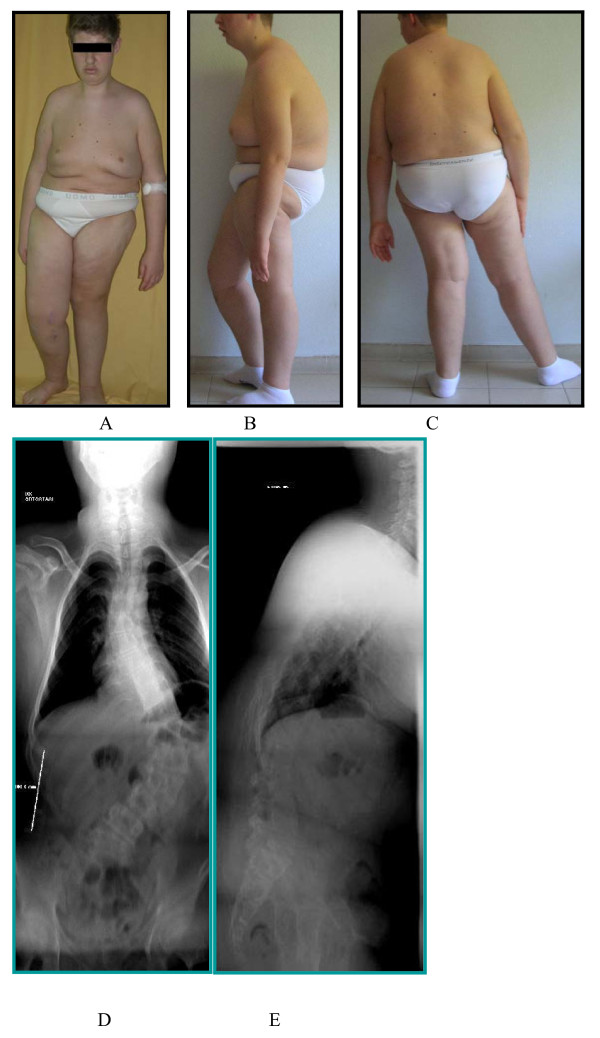
**Preoperative clinical and radiographic picture**. O.M., male, born in 1992, operated on in 2007; severe kyphoscoliosis in PWS in a patient affected with valgus knee deformity.

**Figure 4:**
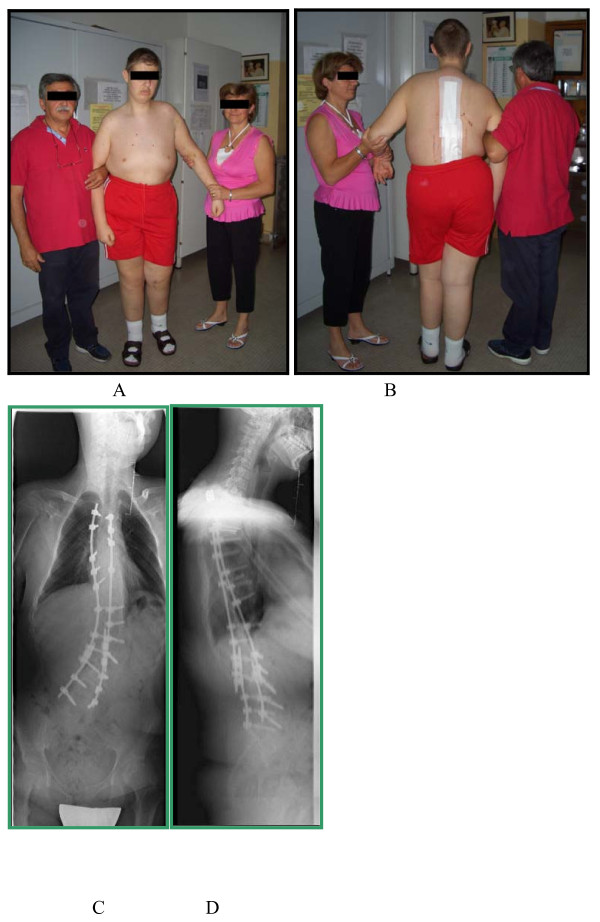
**Clinical and radiographic picture at a 6-month follow-up**. After performing revision surgery and extending arthrodesis to L5, correction was stable.

O.M. (male, born in 1992) The last patient with spinal deformity in P.W. syndrome had received pharmacologic GH treatment for several years and by the age of 15, he presented with marked obesity and a BMI value of 33. During early paediatric age, the patient had undergone surgery for bilateral cryptorchidism and tibial epiphysiodesis for bilateral valgus knee deformity. Strabismus, bilateral nystagmus and lower limb asymmetry were registered, too. The child had a history of fever convulsions until the age of 6 and had received pharmacological treatment with growth hormone for several years. Pulmonary assessment showed a severe deficit with FVC = 46% and FEV1 = 43%. Spine surgery was performed in June 2007, at the age of 15, while the Risser sign was 4. Preoperative radiographic evaluation showed a right convex thoracic scoliotic primary curve of 80° associated with a left lumbar structured compensatory curve of 40°. A marked imbalance could be seen on sagittal plane, since the cervico-sacral vertical plumb line was +15 cm [Figures 3a, 3b, 3c, 3d and 3e]. Instrumented posterior arthrodesis from T5 to L3 using pedicle screws provided satisfactory corrections, as the main thoracic curve decreased to 50°, the secondary lumbar curve to 26° and the sagittal plumb line to +3.8 [Figures 4a, 4b, 4c and 4d]. Operation lasted 6 hours and blood loss was 1000 ml. Six months after surgery, the distal part of the instrumentation underwent revision due to screw loosening in L3. Distal instrumentation was extended to L5. Eighteen months after the second procedure, correction was stable and no complications were observed. However, the child experienced worsening of quality of life due to continuous increase in body weight and deterioration of lung function.

## Results

A total of 6 patients (5 males, 1 female) with scoliosis in PWS were operated on between 1999 and 2007 at the authors' Spinal Surgery Division. Most of the patients showed marked obesity; none of them had congenital vertebral anomalies. Mean age at scoliosis diagnosis was 6.2 years (range 0.5 to 13.5). The primary treatment was bracing although patients' difficulty in tolerating braces often resulted in non-compliance with treatment. However, the outcome in those patients who wore brace full-time was not satisfactory, since the conservative treatment failed to control progression of deformity. Intraoperative neuromonitoring was performed using the Stagnara wake-up test before 2000 and the Somatosensory Evoked Potentials afterwards (associated with Neurogenic Motor Evoked Potentials). Postoperatively, immobilization with brace was prescribed in 3 cases. The mean follow-up was 3 years and 10 months (range 2 years to 9 years). The preoperative mean Cobb angle of primary curves varied from 65° to 96°, while the preoperative kyphosis angle from 31° to 69°.

Three major complications (50%) were encountered: a transient paraparesis, a distal screw loosening and a case of postoperative cervico-thoracic kyphosis. This finding is consistent with the literature, where surgery is reported to be associated with a high rate of complications due to the specific features of this syndrome, such as the ligamentous laxity and natural tendency to maintain a forward-flexed cervical posture, which can be enhanced by excessive halo traction or long Milwaukee brace treatment [8,9].

In case 2 the intraoperative paraparesis, revealed by the Stagnara wake up test (no spinal cord monitoring was performed at the authors' division at that time), required removal of the instrumentation. At follow-up, neurological recovery was near complete but the exact cause of the transient lesion and its exact level could not be assessed.

Screw pullout was observed at caudal level (L3) in case 6; during revision surgery, 3 distal screws were replaced and instrumentation was extended by 2 levels.

In case 4, a cervico-thoracic kyphosis was observed 1 year after instrumenting the patient from T3 to L3 with a growing rod: 3 years after final arthrodesis, the instrumentation was still stable.

Good fusion was achieved in all of the cases and no pseudarthrosis was observed.

No infections were encountered in the present series. Moreover, the lower postoperative pain sensitivity, which is well described in the literature, was confirmed by the current study. Mean surgery time was 5 hours (range, 3 to 6 hours) and mean blood loss was 1750 ml (range, 1000 to 3000).

## Discussion

The literature dealing with scoliosis surgery in PWS consists of only few case reports with a relatively small number of patients [9-15]; generally speaking, the onset of PWS, its curve pattern, the course of progression, guidelines for treatment and surgical indications have been reported to resemble those of the adolescent idiopathic scoliosis [8].

Bracing, as a treatment for scoliosis, remains controversial. Kroonen at al [8] have stated that, although guidelines for brace treatment of scoliosis in PW patients are the same as those of AIS, effective brace moulding is difficult because of the patients' obesity. Remodelling is therefore often necessary on account of patient's weight fluctuations and compliance with the brace protocol depends on the subject's behavioural profile. Accadbled et al [9] have asserted that Milwaukee bracing should be avoided in Prader-Willi patients who have undergone spinal surgery for scoliosis, as it could lead to severe cervicothoracic collapse, eventually requiring reoperation. Three patients of the present series could not tolerate bracing and provided high resistance against it. In the remaining 3 cases, however, progression of deformity did not respond to full-time conservative treatment.

## Conclusions

There is no definite consensus on the indications of surgical correction and instrumentation in PWS scoliosis. As in the case of idiopathic scoliosis, unbalanced and progressive curves represent indications for surgery. However, bracing is often less efficient than in idiopathic scoliosis in halting curve progression because of the patients' obesity and frequent lack of compliance, thus increasing the need for surgical treatment. Despite the fact that spine deformity in PW syndrome may resemble the idiopathic patterns, cardiopulmonary impairment in PW syndrome may occur with kyphoscoliosis less than 100° because of the associated neuromuscular weakness or obesity [16].

The evolution of surgical procedures with modern spinal instrumentation and advanced anaesthesia techniques has enabled significant corrections of spinal deformities to be achieved. The single case of the use of the growing rod technique does not provide sufficient data to make any recommendation for or against its use in PWS. Further studies are required to access its efficacy in younger PWS patients. One has to bear in mind the low stature in PWS and the potential effects of GH treatment.

The use of pedicle screws and advances in anaesthesia techniques [17,18] has improved care for these PWS patients with spine deformities. Instrumentation hybrid constructs and all posterior pedicle screws, and posterior arthrodesis allow surgeons to obtain a stable correction of the deformity. In addition, these new techniques improve the postoperative course by allowing for immediate mobilization without any orthosis.

In the current series, one major neurological complication was encountered consisting in a transient paraparesis. With the reported neurological complications in PWS undergoing deformity surgery we recommend preoperative spinal MRI [19] and spinal cord monitoring during surgery [20].

## Competing interests

The authors declare that they have no competing interests.

## Authors' contributions

TG performed surgeries as first surgeon, conceived the study and coordinated the preparation of the manuscript; MDS performed surgeries as first surgeon; AC performed surgeries as first surgeon; GB participated in the design of the study; KM helped to draft the manuscript; FL did research and helped to list references; GBB kept contacts with patients and assembled data; SG helped in data analysis. All authors read and approved the final manuscript.

## Authors' information

TG is also the Head of a highly-specialized outpatient surgery unit at the Rizzoli Orthopaedic Institute, Bologna (I), dedicated to the study and treatment of severe spinal deformities.
